# Adenylylsulfate–ammonia adenylyltransferase activity is another inherent property of Fhit proteins

**DOI:** 10.1042/BSR20150135

**Published:** 2015-08-07

**Authors:** Anna M. Wojdyła-Mamoń, Andrzej Guranowski

**Affiliations:** * Department of Biochemistry and Biotechnology, Poznań University of Life Sciences, 60–632 Poznań, Poland

**Keywords:** adenosine 5′-phosphoramidate, adenosine 5′-phosphorofluoridate, adenosine 5′-phosphosulfate, fragile histidine triad protein (Fhit)-catalysed ammonolysis, fragile histidine triad (Fhit) proteins

## Abstract

Fhits (fragile histidine triad proteins) occur in eukaryotes but their function is largely unknown, although human Fhit is believed to act as a tumour suppressor. Fhits also exhibit dinucleoside triphosphatase, adenylylsulfatase and nucleoside phosphoramidase activities that in each case yield nucleoside 5′-monophosphate as a product. Due to the dinucleoside triphosphatase activity, Fhits may also be involved in mRNA decapping. In the present study, we demonstrate Fhit-catalysed ammonolysis of adenosine 5′-phosphosulfate, which results in the formation of adenosine 5′-phosphoramidate. This reaction has previously been associated with adenylylsulfate–ammonia adenylyltransferase (EC 2.7.7.51). Our finding shows that the capacity to catalyse ammonolysis is another inherent property of Fhits. Basic kinetic parameters and substrate specificity of this reaction catalysed by human Fhit are presented.

## Abstract (precis)

Activity of an alleged adenylylsulfate–ammonia adenylyltransferase is actually an inherent property of fragile histidine triad proteins (Fhits). We describe how our search for the transferase led to this serendipitous finding and provide basic kinetic parameters of ammonolytic reactions catalysed by human Fhit.

## INTRODUCTION

Fragile histidine triad proteins (Fhits) attract interest of biologists and biochemists over the last two decades. (On the PubMed site, one can find today over 1000 Fhit-related articles). Mammalian Fhit functions both as tumour suppressor and genome ‘caretaker’ [1]. The human Fhit was shown to be a typical dinucleoside triphosphate hydrolase (EC 3.6.1.29) [2] that catalyses reaction 1 (Table 1). Among good substrates of dinucleoside triphosphatases/Fhits are mRNA 5′-cap analogues [2–4] and this explains why Fhits may also function as mRNA decapping enzymes [5,6]. The present paper describes sequence of events that led to the discovery of novel catalytic capacity of Fhits.

**Table 1:** Reactions catalysed by HIT-proteins, mostly by Fhits, considered in the present article

Reaction number	Reaction equation	References
1	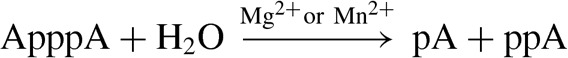	[2]
2	NH_2_-pA + H_2_O → pA + NH_3_	[7–10]
3*	NH_2_-pA + P_i_ → ppA + NH_3_	[11]
4	SO_4_-pA + NH_3_ → NH_2_-pA + SO_4_^2−^ + H^+^	present paper
5	F-pA + NH_3_ → NH_2_-pA + F^−^	present paper
6	ApppA + NH_3_ → NH_2_-pA + ppA	present paper
7	F-pA + H_2_O → pA + F^−^	[7]
8	SO_4_-pA + H_2_O → pA + SO_4_^2−^ + H^+^	[7,8]
9	SO_4_-pA + F^−^ → F-pA + SO_4_^2−^	[21]
10	NH_2_-pA + F^−^ → F-pA + NH_3_	[21]

*Fhits do not catalyse this phosphorolytic reaction.

Our long-lasting interest in enzymes involved in the metabolism of minor nts has recently focused on enzymes that catalyse degradation of adenosine 5′-phosphoramidate (NH_2_-pA), either hydrolytically to 5′-AMP (pA) and ammonia [7–10] (Table 1, reaction *2*) or phosphorolytically to 5′-ADP (ppA) and ammonia [11] (reaction 3). Proteins exhibiting these activities belong to the HIT superfamily of proteins that contain histidine-triad sequence motif and occur in all type of cells. NH_2_-pA was discovered in 1981 among nts purified from the extracts of green alga (*Chlorella pyranoidosa*) cells [12] and proved to be a product of (reaction 4) catalysed by an enzyme that had been called adenylylsulfate–ammonia adenylyltransferase and classified under the entry EC 2.7.7.51. This transferase activity was identified in different organisms including higher plants, barley and spinach and the relevant protein purified from *Chlorella* extracts [13]. Since then, however, studies on this enzyme and on the metabolic fates of NH_2_-pA have been neglected. We wanted to study an adenylylsulfate–ammonia adenylyltransferase protein and wondered if it also occurs in yellow lupin seeds, used by us as a good source of many enzymes of basic nucleoside and nt metabolism, as well as enzymes involved in the catabolism of minor di- and mono-nts, such as diadenosine triphosphate (ApppA) [3,14], diadenosine tetraphosphate (AppppA) [14], adenosine 5′-tetraphosphate (ppppA) [15] or the aforementioned NH_2_-pA [9]. In pilot experiments with lupin extracts, we did find an activity that catalysed reaction 4. The developed purification procedure (see below) yielded a homogeneous protein preparation exhibiting adenylylsulfate–ammonia adenylyltransferase activity. Analysis of the data obtained by MALDI–TOF MS serendipitously revealed a similarity between this lupin protein and a plant (chickpea, *Cicer arietinum*) dinucleoside triphosphatase that is known to catalyse reaction 1. This was a turning point in our study. Knowing that this latter activity is a feature of various Fhit proteins [2,8,16], we checked whether recombinant Fhits from humans, *Trypanosoma brucei* or *Arabidopsis thaliana* [8] could also catalyse the ammonolytic reaction and it appeared that they indeed could. Below, we characterize the ammonolytic reactions catalysed by recombinant human Fhit. (Preliminary communication on this novel catalytic capacity of Fhits was presented at the 49^th^ Meeting of the Polish Biochemical Society [17]).

## MATERIALS AND METHODS

### Biological material

Yellow lupin (*Lupinus luteus*) seeds were from the Plant Breeding Station in Wiatrowo near Poznań, Poland. Origin of the recombinant *T. brucei* [16], human and *A. thaliana* Fhits and procedures used for their purification were described previously [7].

### Reagents

CAPS [3-(cyclohexylamino)-1-propanesulfonic acid], NH_2_-pA, common adenine nts, other nucleoside 5′-monophosphates and basic chemicals were from Sigma. Nucleoside 5′-phosphosulfates (SO_4_-pNs), including adenosine 5′-phosphosulfate (SO_4_-pA and adenosine 5′-phosphosulfate-3′-phosphate), were synthesized according to Kowalska et al. [18]. Adenosine 5′-phosphorofluoridate (F-pA) was synthesized according to Wittmann [19]. Custom labelled NH_2_-p[8-^3^H]A and F-p[8-^3^H]A were purchased from MoravekBiochemicals. Standards of guanosine-, cytidine- and uridine-5′-phosphoramidates were kindly donated by Professor Adam Kraszewski and Dr Joanna Romanowska (Institute of Bioorganic Chemistry, Polish Academy of Sciences in Poznań).

### Buffers

Buffer A: 20 mM potassium phosphate (pH 6.8) containing 5% glycerol and 1 mM 2-mercaptoethanol; buffer B: 50 mM potassium phosphate (pH 6.8) containing 5% glycerol and 1 mM 2-mercaptoethanol; buffer C: 50 mM Tris/HCl (pH 8.0) containing 5% glycerol and 1 mM 2-mercaptoethanol.

### Enzyme assays

The mixture (25 μl of final volume) used for monitoring adenylyl sulfate–ammonia adenylyltransferase activity during purification of the lupin enzyme contained 500 mM NH_4_HCO_3_, 1 mM SO_4_-pA or 1 mM F-pA and the analysed fraction. Incubation was carried out at 30°C. After the appropriate time, depending on the purification stage, 3 μl aliquots of the mixtures were spotted on a TLC plate (aluminium pre-coated with silica gel containing fluorescent indicator, from Merck). The chromatogram was developed for 30–40 min in 2-propanol–25% ammonia–water [11:2:2 (v/v)] and nt spots visualized under shortwave UV light. In active fractions, the substrate was converted to NH_2_-pA. This qualitative approach was sufficient for making quick decisions about which fractions from one step of enzyme purification could be collected and used for a following step. Slightly modified assays are described in the legends to Figures 1 and 2.

**Figure 1:**
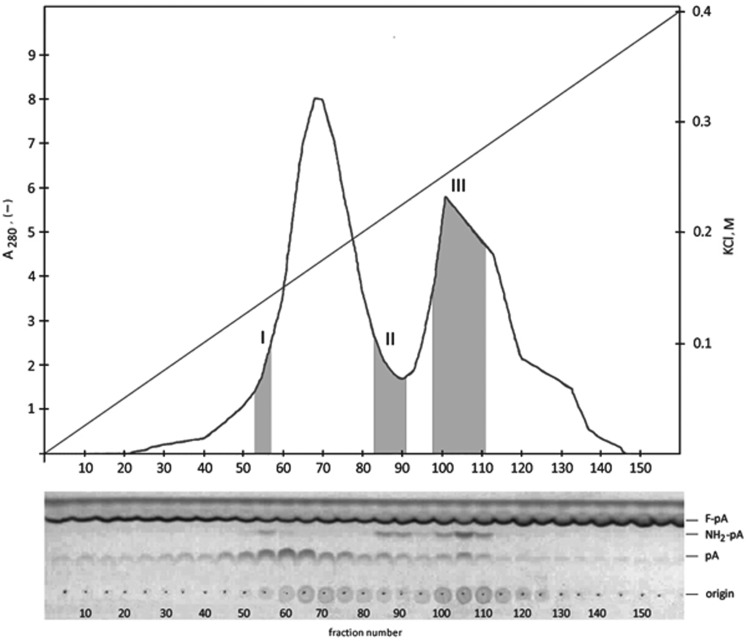
Purification of yellow lupin adenylylsulfate–ammonia adenylyltransferase on DEAE-Sephacel column (5×15 cm) and TLC analysis of the enzyme activity of the reaction mixtures with F-pA as an alternative substrate of ammonolysis The analysis presented involved samples that were incubated for 2 h.

**Figure 2:**
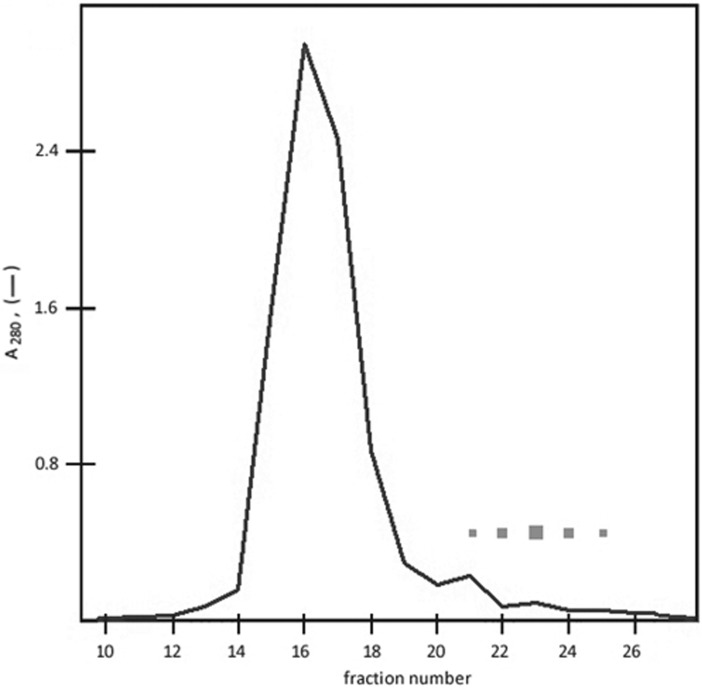
Purification of yellow lupin adenylylsulfate–ammonia adenylyltransferase by gel filtration on a Sephadex G-100 column (1.6×90 cm) The squares represent the locations of active fractions.

Rates of ammonolysis of unlabelled substrates were analysed by TLC and densitometry of reaction product spots by the use of the G:Box system and the Gene Tools program. Within the range of 0.5 and 1.5 nmol of nt per spot, there was perfect linear dependence of attenuance compared with nt amount [10].

### Estimation of *K*_m_ values

*K*_m_ for SO_4_-pA in the ammonolytic reaction was carried out in mixtures (25 μl) containing 200 mM CAPS/NaOH (pH 10.6), 185 mM NH_3_, SO_4_-pA in the range 0.3–1 mM and rate-limiting amounts of human Fhit (0.03 μg). At time intervals 0, 10, 20, 30 and 40 min, 3 μl of aliquots were spotted at the origin of a chromatographic plate, 2 nmol NH_2_-pA standard added and the plate developed for 30 min in 2-propanol–25% ammonia–water [11:2:2 (v/v)]. Nt spots were visualized under shortwave UV light. Initial rate for each substrate concentration was estimated by densitometry (see above).

Estimation of *K*_m_ for F-pA in the ammonolytic reaction was carried out in mixtures (25 μl) containing 200 mM CAPS/NaOH (pH 10.6), 185 mM NH_3_ and labelled F-p[^3^H]A in the range 0.62–5 mM and rate-limiting amounts of human Fhit (0.03 μg). At time intervals 0, 20, 40, 60 and 120 min, 3 μl of aliquots were spotted at the origin of a chromatographic plate, 2 nmol NH_2_-pA added and the plate developed for 30 min in 2-propanol–25% ammonia–water [11:2:2 (v/v)]. Nt spots were visualized under shortwave UV light. Spots of labelled NH_2_-pA were excised, immersed in scintillation cocktail and radioactivity determined. Radioactivity in 3 μl of aliquots ranged between 60000 and 430000 cpm.

Estimation of *K*_m_ for NH_3_ in the ammonolysis of F-p[^3^H]A was carried out in mixtures containing 200 mM CAPS/NaOH (pH 10.6), 1 mM F-p[^3^H]A, NH_3_ in the range 5.7–153 mM and rate-limiting amounts of human Fhit (3 μg). To estimate initial rates for each concentration of NH_3_, 3 μl of aliquots were withdrawn at time intervals 0, 10, 20, 40 and 80 min, spotted at the origin of a chromatographic plate and 2 nmol NH_2_-pA added. The plate was developed for 30 min in 2-propanol–25% ammonia–water [11:2:2 (v/v)] and nt spots visualized under shortwave UV light. Spots containing labelled NH_2_-pA were excised, immersed in scintillation cocktail and radioactivity determined. Radioactivity in 3 μl of aliquots was 85000 cpm. In each case, the *K*_m_ values were calculated from Eadie–Hofstee plots (*v* compared with *v*/[S]).

### Electrophoresis

SDS/PAGE was conducted in 16% gels and native PAGE in 12% gels according to Laemmli [20]. After native electrophoresis, the lane with the enzyme sample was cut, gel strips were incubated in the standard reaction mixture and the effects analysed by TLC as described above.

### MALDI–TOF mass spectrometric analysis

This was performed at the proteomics facility of the Institute of Biochemistry and Biophysics, Polish Academy of Sciences in Warsaw.

## RESULTS AND DISCUSSISON

### Purification of adenylylsulfate–ammonia adenylyltransferase from yellow lupin seeds

Meal (200 g), obtained from yellow lupin seeds, was extracted with 700 ml of buffer A. The slurry was centrifuged (15 min at 20000 ***g***) and the transferase activity was precipitated from the resulting supernatant with ammonium sulfate (50%–70% saturation). The precipitate was dissolved in buffer B and the resulting solution dialysed against this buffer. After centrifugation (15 min at 20000 ***g***), the dialysate was applied to a DEAE-Sephacel column (5×15 cm) equilibrated with the same buffer. The column was washed with 1.5 l of buffer B followed by a linear gradient of 0–0.5 M KCl in the same buffer (total 3 l). Of the three active peaks (Figure 1), fractions of peak III, eluted at 0.15–0.25 M KCl, were pooled and subjected to concentration by ultrafiltration (AMICON ULTRA 30 filters). The resulting sample (1.5 ml) was subjected to gel filtration on a Sephadex G-100 column (1.6×90 cm) equilibrated with buffer C and 4 ml of fractions were collected. The transferase activity emerged in fractions 21–25 (Figure 2). These fractions were pooled and applied to a 1 ml column of AMP-agarose (Sigma) equilibrated with buffer C. After washing with buffer C (6×1 ml) and with buffer C containing 250 mM KCl (5×1 ml), the enzyme was eluted with the same solution containing 5 mM AMP. Active fractions were concentrated by ultrafiltration (AMICON ULTRA Ultracel 10K) with simultaneous buffer exchange back into buffer C without additions.

### Purity of the lupin transferase

As shown in Figure 3, the enzyme preparation exhibited a single protein band of approximately 18 kDa. This is the mass of the monomer, as its position on the gel filtration elution profile indicated that the native enzyme functions as an α_2_ dimer of 36 kDa. Figure 4 shows also a single band on the native gel. Incubation of strips from a parallel lane revealed that the protein band retained adenylylsulfate–ammonia adenylyltransferase activity.

**Figure 3:**
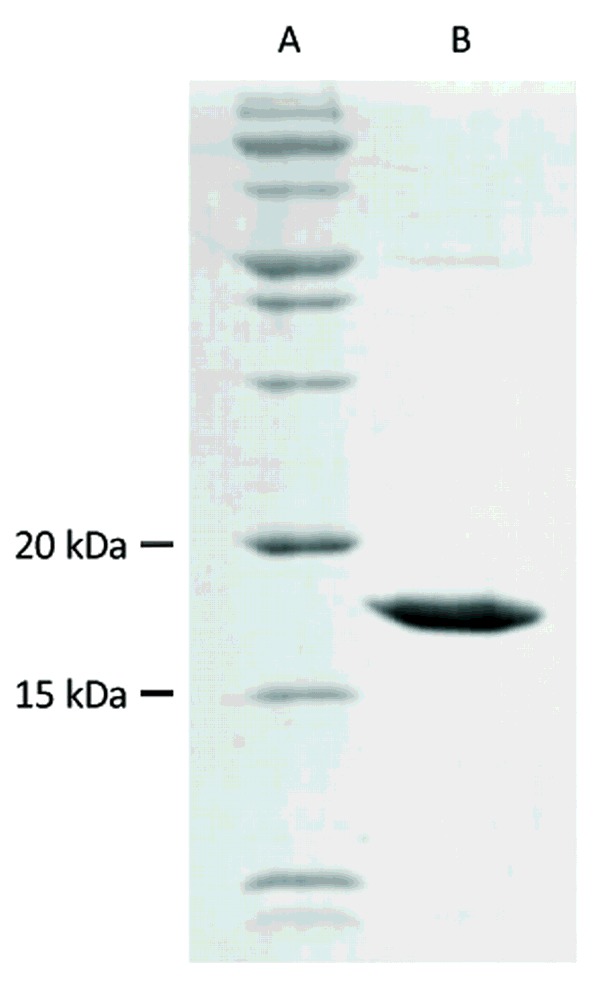
Purity of the yellow lupin adenylylsulfate–ammonia adenylyltransferase after affinity elution from AMP-agarose demonstrated by SDS/PAGE Lane A, molecular mass standard proteins; lane B, 5 μg of the purified enzyme.

**Figure 4:**
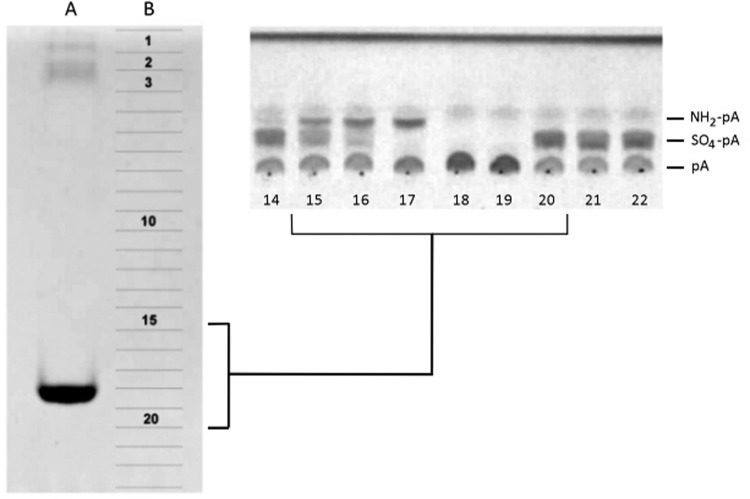
Native PAGE of the yellow lupin adenylylsulfate–ammonia adenylyltransferase and identification of the enzyme activity in gel strips The enzyme activity was assayed as described in ‘Materials and Methods’.

### Short comment on the enzyme purification

Protein that exhibited activity of adenylylsulfate–ammonia adenylyltransferase has been purified to homogeneity by simple, conventional techniques. It is worth noting, however that of the four different AMP-agaroses, only the gel from Sigma, with AMP attached to the matrix at its N^6^ via an eight-atom-spacer, selectively adsorbed the enzyme and released it when subjected to elution with buffer containing 5 mM AMP.

### Mass spectrometric analysis

Protein from the gel shown in Figure 3 was subjected to digestion with trypsin and MALDI–TOF mass spectrometric analysis. Peptides that corresponded to the protein of the highest score were then submitted to a BLAST search. The lupin protein revealed a similarity to a plant (*Cicer arietinum*, chickpea) dinucleoside triphosphatase.

This serendipitous finding switched our study towards various Fhit proteins that have been known as dinucleoside triphosphatases (EC 3.6.1.29). We demonstrated that recombinant human and *A. thaliana* Fhits and *T. brucei* HIT-45 protein do catalyse ammonolysis of SO_4_-pA. Detailed studies on this reaction were carried out with the recombinant human Fhit.

### Ammonolytic reactions catalysed by human Fhit

As shown in Figure 5, human Fhit catalysed, in addition to ammonolysis of SO_4_-pA, reaction 4, the ammonolysis of adenosine 5′-phosphorofluoridate (F-pA), reaction 5 and of diadenosine triphosphate (ApppA), reaction 6. It was also shown that these conversions were Fhit-dependent. In addition to increasing amounts of NH_2_-pA in the incubations containing SO_4_-pA or F-pA, we observed the slow accumulation of AMP (pA), which is a product of the Fhit-catalysed hydrolysis of NH_2_-pA, reaction 2 and F-pA, reaction *7* [7]. In the lanes involving incubations with ApppA, another product of reaction 6, ppA, remained at the origin. Based on densitometric measurements, the relative velocities of these three reactions were 100:2.6:0.16 respectively.

**Figure 5:**
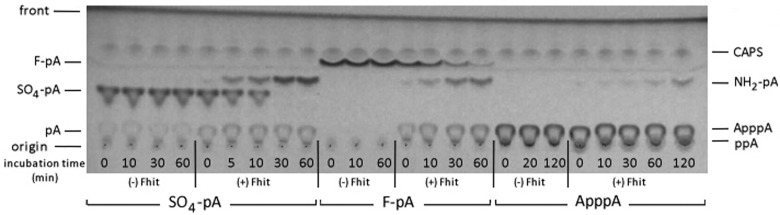
Time-course of Fhit-dependent ammonolysis of adenosine 5′-phosphosulfate, adenosine 5′-phosphorofluoridate and diadenosine triphosphate Reaction mixtures (25 μl) contained 200 mM CAPS/KOH (pH 10.6), 500 mM NH_3_, 1 mM indicated substrate and either 0.2 μg of recombinant human Fhit in the case of SO_4_-pA or 1 μg in the case of F-pA or ApppA. Incubation was carried out at 30°C. At the times indicated, 3 μl aliquots of the mixtures were spotted on a TLC plate (aluminium pre-coated with silica containing fluorescent indicator, from Merck). The chromatogram was developed for 40 min in 2-propanol–25% ammonia–water [11:2:2 (v/v)] and photographed under short-wave UV light. Each substrate was also incubated in the same buffer in the absence of Fhit to check its stability under the assay conditions.

We also found that human Fhit could catalyse the ammonolysis of all the 5′-phosphosulfates (SO_4_-pNs) of the four canonical ribonucleosides (Figure 6). Rates were again estimated by densitometry. Setting the preferred substrate, SO_4_-pA, as (100), relative rates were: SO_4_-pC (91), SO_4_-pG (47) and SO_4_-pU (56). Kinetic parameters of the ammonolytic reactions 3 and 5 were as follows: *K*_m_ for SO_4_-pA, 2.4±0.3 mM; *K*_m_ for F-pA, 1.6±0.2 mM; and *K*_m_ for NH_3_ estimated for the ammonolysis of F-pA, 60±15 mM. The hydrolytic reactions 2, 7 and 8 had pH optima between 6.5 and 7 [7,21]. In reactions containing 500 mM ammonium bicarbonate (pH 9), rates were about 20 times slower than at neutral pH, with no reaction proceeding in mixtures containing CAPS and NH_3_ at a pH of around 10.6. (Compare the lanes involving SO_4_-pA in Figure 6 compared with Figure 5 respectively). It is worthy adding that adenosine 5′-phosphosulfate-3′-phosphate, SO_4_-pAp (known as PAPS), was not a substrate of the Fhit-catalysed ammonolysis.

**Figure 6:**
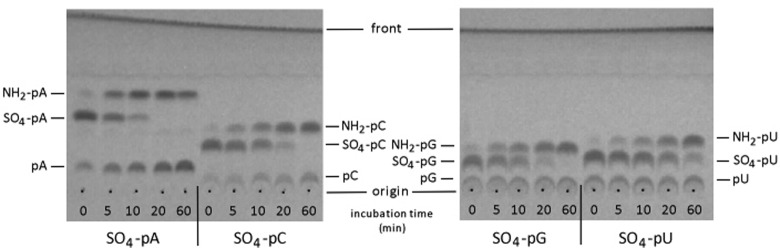
Time-course of Fhit-dependent ammonolysis of various nucleoside 5′-phosphosulfates analysed by TLC The reaction mixture (25 μl) contained 500 mM NH_4_HCO_3_, 1 mM indicated SO_4_-pN and 0.1 μg of human Fhit. The time-course of the reaction was analysed by TLC as described in the legend to Figure 1, except that the chromatogram was developed for 75 min. The original chromatogram was cut in half to facilitate description of the spots.

A possible mechanism for Fhit-catalysed ammonolysis of these substrates most probably involves attack by NH_3_ on the covalent histidine-bound enzyme-nucleosidyl intermediate which has previously been demonstrated for Fhit in studies on the mechanism of its action on phosphorus-chiral substrates [22,23]. At high pH and high concentration of NH_3_, the intermediate can be efficiently intercepted even in the presence of solvent water leading to the formation of a nucleoside 5′-phosphoramidate (NH_2_-pN).

In conclusion, our finding shows first of all that the activity of an alleged adenylylsulfate–ammonia adenylyltransferase is actually an inherent property of Fhits and secondly, adds a new facet to the catalytic promiscuity of an enzyme that so far has been shown to catalyse reactions assigned as dinucleoside triphosphate hydrolase (EC 3.6.1.29), reaction 1, adenylylsulfate sulfohydrolase (EC 3.6.2.1), reaction 8 and nucleoside phosphoramidate hydrolase (EC 3.9.1.-), reaction 2. (The Enzyme Commission has not yet classified phosphoramidases that are specific for nucleoside phosphoramidates. Therefore, these hydrolases await their fourth digit). Very recently, Fhits have been demonstrated to catalyse also fluorolysis of nucleoside 5′-phosphoramidates and nucleoside 5′-phosphosulfates, reactions 9 and 10 [21]. Whether the reported Fhit-mediated ammonolysis of certain nts remains only a curiosity or has some biological relevance requires further studies. Anyhow, our finding should now be taken into account in studies of basic metabolism, in particular of the sulfur assimilation, SO_4_-pA conversions and the biological role of Fhits. Speculating on this issue, we see at least the following ways by which the novel Fhit capacity could affect general metabolism: Fhits may compete for and control the cellular level of SO_4_-pA which in the reaction catalysed by adenylylsulfate kinase (EC 2.7.1.25) is converted to SO_4_-pAp, the main donor of sulfate moiety to polysaccharides or various low-molecular compounds. In plants, SO_4_-pA can be a substrate of the adenylylsulfate reductase (EC 1.8.4.9) that catalyses the glutathione-involving reaction leading to sulfite [24]. Fhit may also function as a cellular switch that, depending on availability of substrates, forms NH_2_-pA in the revealed reaction 4 or hydrolyses it in reaction 2. Thus Fhits may be involved in distribution (channelling) of ammonia; the molecule that, in addition to conversions on the main metabolic pathways involving carbamoyl phosphate biosynthesis, has been recognized as a diffusible regulator of autophagy in human cells [25]. Finally, NH_2_-pA should be considered as a novel signal molecule. Recently, this idea has gained support from experiments performed in our laboratory on *Arabidopsis* seedlings [26]. Micromolar concentration of exogenous NH_2_-pA added to the growth medium induced in these seedlings activity of several genes that encode enzymes catalysing key reactions of phenylpropanoid pathways and caused accumulation of lignins, anthocyanins and salicylic acid, the compounds that appear in the plant cells in response to stress. Future experiments will answer the question whether NH_2_-pA behaves as a signal molecule also in mammalian and human cells.

## Abbreviations

CAPS: 3-(cyclohexylamino)-1-propanesulfonic acid
Fhit: fragile histidine triad protein
F-pA: adenosine 5′-phosphorofluoridate
HIT proteins: superfamily of proteins that contain histidine-triad sequence motif
NH_2_-pA: adenosine 5′-phosphoramidate
SO_4_-pA: adenosine 5′-phosphosulfate
SO_4_-pN: a nucleoside 5′-phosphosulfate
